# Growing third places: A qualitative study of experiences and perceived benefits of a campus community garden as a nature-based health intervention

**DOI:** 10.1371/journal.pone.0338602

**Published:** 2025-12-31

**Authors:** Olivia D’Andrea Brooks, Rubiga Thanganathan, Lesley Gittings

**Affiliations:** 1 School of Health Studies, Western University, London, Ontario, Canada; 2 Children’s Health Research Institute, Lawson Health Research Institute, London, Ontario, Canada; 3 Centre for Social Science Research, University of Cape Town, Cape Town, South Africa; Central Queensland University, AUSTRALIA

## Abstract

This study explored the experiences and perceived health and wellbeing benefits of participating in a campus community garden among university staff, students, and faculty. Community gardens are increasingly recognized for their potential to positively impact physical, mental, and emotional health, as well as increase social connection. Their impact within university settings, specifically in Canadian post-secondary contexts, remains underexplored. This qualitative study was conducted at a large Canadian university. Fourteen participants affiliated with the campus community garden (including students, staff, and faculty) participated in semi-structured, in-depth interviews. Data were analyzed using thematic analysis. Four key themes emerged: (1) the garden as a “third place”, (2) deepened connection to nature, (3) empowerment through knowledge and ability to grow food, and (4) community building. Findings suggest that campus community gardens offer a cost-effective and accessible approach to supporting mental wellbeing, food security, and connectedness to nature in post-secondary settings. By functioning as a third place and fostering engagement with nature, campus community gardens may provide students and staff with meaningful opportunities for community integration, improved wellbeing and personal growth. These findings support integrating community gardens into campus wellness strategies and sustainability initiatives.

## 1. Introduction

Nature-based health interventions (NBHI) are broadly defined as activities, programs or strategies that facilitate engagement in nature-based experiences with the aim to improve aspects of health and well-being [1]. NBHI aim to change human behaviour (e.g., encouraging exercise in nature, ecotherapy) or change the environment (e.g., create gardens or public parks) [2]. A key aim of NBHI is to foster human connectedness to nature to improve well-being [3]. A scoping review on NBHI for vulnerable youth grouped NBHI into four categories: wilderness-therapy interventions, animal-assisted interventions, care farming interventions, and gardening and horticulture-based interventions [4]. Gardening and horticultural interventions center on gardening and horticultural activities, such as planting and maintaining a community garden plot or food orchards [4,5]. These interventions not only encourage physical activity through manual tasks like digging, weeding, and harvesting, but may also promote psychological well-being by fostering mindfulness, reducing stress, and offering a sense of accomplishment and connection to the natural world [6].

Community gardens as a form of NBHI offer notable benefits such as enhancing physical [1], mental, and emotional health and wellbeing [7,8], increasing connection with nature [5], alleviating food security issues [9], and building community among participants [10]. The physical and mental health and well-being benefits are among the more well explored and reported impacts of participation in community gardens [11,12]. Participation in community gardens can improve knowledge about nutrition, increase physical activity and reduce body weight and hypertension [10]. Additionally, participation in community gardens can also reduce stress [10,11] which can positively impact participants’ cardiovascular health, among other physical health benefits [13]. Further, the connection between nature and human well-being is increasingly recognized, including psychological and physical health benefits such as alleviation of anxiety and depression and improved cardiovascular function [14–18]. Other mental health and well-being benefits from participation in community garden initiatives may include improvements in self-esteem, joy, relaxation, and independence [19,20]. Participation in community gardens provides an alternative means of connecting with nature and can help put life into perspective, giving participants a sense of pride and purpose [21]. Community gardens can also contribute to community building and the development of social capital by bringing people with a common purpose in a shared activity together [10].

Campus community gardens can serve as practical venues for applied learning and research, often incorporated into academic programs on college and university campuses such as horticulture, landscape design, and sustainability studies [22]. Campus community gardens offer both social and educational benefits, playing an important role in heightening a sense of environmental responsibility [22,23]. Further, they provide a platform for engaging the university community in addressing issues such as mental health and food security [22,24]. The importance of this cannot be overstated, as post-secondary institutions are increasingly recognized not only as high-stress environments, but also as important grounds for promotion of mental health and well-being [25,26].

Community gardens as NBHI thus present an opportunity to serve as a cost-effective, multi-faceted health intervention, as well as an activity that builds community on campus [2]. Despite their potential, the integration, utilization, and impact of community gardens within university environments have not been extensively examined, and this is especially true in the Canadian context. This study aimed to address this gap, by: (1) exploring and documenting the experiences of participation in a campus community garden; and (2) identifying and understanding the perceived health and well-being benefits of garden participation.

## 2. Materials and methods

We conducted a qualitative study in 2023–2024 on the Western University campus community garden in London, Ontario, Canada. There are 38 total plots (raised concrete planter beds for food crops), with about 14 plots being allocated to two different campus organizations to distribute amongst their members (consisting of undergraduate and graduate students). There are 21 additional planters on the perimeter of the garden for native plants to support pollination and biodiversity [27]. There are no specific allocation requirements for students, staff, or faculty members. The remaining 24 plots are distributed based on a lottery system prior to the beginning of the summer growing season. At the time of research, participants were oriented to the garden by an initial group planting day where all participants attended in person and were offered a tour of the space, guidelines for using the hose, how to dispose of plant waste, etc. They were not offered any guidance on what or how to grow at that time.

Data were collected through in-depth, semi-structured interviews (Table 1) ranging from 20−90 minutes, with most interviews being 40–65 minutes. The total number of garden participants for the two years from which recruitment occurred was 163 people, with an approximate breakdown of total participants in the garden being 75% students, 15% staff, and 10% faculty. Fourteen people who had participated in the Western community garden enrolled as research participants, representing a range of affiliations, with some holding multiple roles within the community. Participants included staff and faculty members (n = 9), graduate students (n = 7), undergraduate students (n = 1) and London community members (n = 1). Members from two campus organizations were represented, including a graduate students association (n = 5) and a campus garden association (n = 2). Participants were women (trans-inclusive) (n = 8), men (trans-inclusive) (n = 5), and Two-Spirit individuals (n = 1). Participants were asked to identify as students, staff, faculty, and/or community members based on how they viewed their relation to the community garden. While there were no external community members participating, one participant viewed their connection to the garden first and foremost as a community member. Ethics approval was received from Western University Office of Non-Medical Research Ethics Board (2023-123609-84772). Following ethics approval, recruitment took place from November 6, 2023, until March 20, 2024. Criteria for participation included participants being 18 years of age or older and having participated in the university community garden within the past three years. Due to the qualitative nature of this study and the relatively small, identifiable community from which participants were recruited, we are unable to make full interview transcripts publicly available. Sharing complete qualitative data could risk inadvertent disclosure of participant identities or allow the data to be interpreted out of its original context. To maintain participant confidentiality, we have provided anonymized excerpts within the manuscript that support our findings. Verbal consent was requested while audio recording for Zoom interviews, prior to starting the interview. Participants viewed the screen shared by the researcher as they went through the consent form and were asked to provide verbal consent after being provided with the opportunity to ask any questions. Verbal consent was then recorded electronically on the form (participant name, date, researcher name) by the researcher while the participant viewed the screenshare form. For the in-person interview, the participant read and signed the consent form on their own. Participants were emailed a copy of the information sheet to keep for their own records. Interviews were conducted over Zoom (n = 13) or in a private location selected by participants (n = 1), such as offices or private research spaces on campus. Interviews were audio recorded and transcribed using built-in Zoom software for both Zoom and in-person interviews, with transcriptions being de-identified, checked and transcribed verbatim following the interviews. This study was conducted in partnership with the Western Sustainability Office, Western University in London, Ontario.

**Table 1:** Semi-Structured Interview Guide.

Section	Question	Probe (if applicable)
Introduction	Can you please start by telling us a little about yourself?	What is your year of study or where is your workplace? What is your connectedness to Western University?
Motivations for Participation	How did you first hear about the community garden, and what motivated you to get involved?	Have your motivations evolved or changed over time? If yes, how?
Motivations for Participation	Can you tell us more about your specific role or activities within the university community garden?	How long have you been involved, how often do you visit the garden, did you enrol by yourself or with anyone else?
Motivations for Participation	Are there any specific goals or outcomes you hoped to achieve through your involvement in the community garden?	For example, growing nutritious food, saving money on groceries, getting outdoors, being more environmentally sustainable, or getting involved in the university community?
Perceptions and Experiences	What aspects of the community garden do you find most valuable or rewarding?	
Perceptions and Experiences	Are there any challenges or barriers you have encountered while participating in the community garden?	How have you addressed or coped with them?
Benefits	How has your involvement in the community garden influenced your feelings of connectedness to nature?	Can you please share any specific experiences or moments in the garden that deepened your connection with nature?
Benefits	Do you believe that participating in the community garden has affected your sense of self-efficacy, i.e., the ability to take environmentally sustainable actions?	Have you made any changes in your daily life or behaviours related to sustainability or climate-conscious practices as a result of your experiences in the garden?
Benefits	The community garden could be considered a form of nature-based health intervention. The premise of nature-based health interventions is getting involved in nature, with the hope of experiencing certain positive physical or mental health impacts. In your opinion, has this community garden contributed to your well-being?	Have you experienced any positive emotional or mental health benefits through your involvement in the garden?
Benefits	From your perspective, do you see the university community garden as a potential method to increase food security for undergraduate students?	Why or why not? How can the community garden be utilized to address food insecurity effectively?
Garden Improvements and Changes	In your opinion, what aspects of the community garden could be improved?	Are there any changes you would suggest to enhance the garden’s effectiveness and appeal?
Garden Improvements and Changes	Are there any specific additions or features you believe would be valuable to incorporate into the community garden?	
Garden Improvements and Changes	If you could wave a magic wand and create the best community garden for Western University, what would you do?	What insights or suggestions would you like to share with us to help improve and support the new garden? Accessibility and Inclusivity; Plot Size Preferences; In-Ground Beds vs. Raised Beds; Plot Maintenance and Neglect
Final Thoughts	Is there anything else you would like to share about your experiences with the university community garden or any other insights related to the topics we discussed?	

Data analysis was guided by Braun and Clarke’s (2006) [28] six-step thematic analysis method using an inductive, code-book style analysis [29]. This analytic approach emphasizes a systematic yet flexible process to identifying patterns across a set of qualitative data while maintaining sensitivity to participant meaning. First, authors ODB and RT independently reviewed the transcribed interviews multiple times to familiarize themselves with the data. Following this, initial inductive codes were generated. Codes were then collated into a shared codebook on Microsoft Excel, which was then iteratively refined through discussion to ensure coherence and conceptual clarity. Through this process of reviewing, defining, and naming, broader themes and sub-themes were developed to capture patterns of meaning across the dataset. While this analysis was primarily structured through a shared codebook, it was also informed by reflexive principles in that researchers engaged in ongoing reflection on their interpretations and positionalities throughout the analytic process [29]. Consistent with recent qualitative research guidance, the concept of conceptual sufficiency [30], rather than data saturation was used to determine the adequacy of the dataset.

The thematic analysis culminated in a rich, detailed, and complex account of the data, underscoring the significance and contextual relevance of the study’s findings in relation to the garden and perceived benefits of participation such as connectedness to nature, growing food, and community building.

## 3. Results

Four key themes emerged from the data across participant groups. These included perceived benefits of the campus community garden of 1) the garden as a third place, 2) increased connectedness to nature, 3) empowerment through growing food and, 4) the garden as a facilitator for community building.

### 3.1. The garden as a third place

Many participants spoke about the garden as a third place – a space outside of home and work and/or studies – in different but related ways. The garden served as a physical transition space for some, and for others as a way to bridge the gap between work or studies, and home life. Additionally, it offered a welcoming space that drew participants to campus even when they had no other obligations. The garden reinforced its role as a third place by encouraging voluntary presence, leisure, and informal connection to nature and community.

*“I just knew I wanted to take a break and go for a stroll around the campus and this was the perfect, you know, excuse go down and see what is growing, what needs to be watered. So I found I was certainly going there once a week”* (Staff/Faculty).

*“I don’t necessarily have to go to campus… But I do like having that structure, when I can end and just having that extra push to “all right you need to look after your poor plants.” So it was great for just getting me out there and offering also a bit of a natural breaks so saying, “all right, I do know that I need to water these things today”. So it makes as much sense to just to be out of my way from my apartment, stop at the garden and then be in the office and have that separation to say, “All right, I have left one space. So I’ve taken some time between those spaces and now I’m setting up for my day of work”* (Student).

*“...on the weekends if I needed something to do, I’d come weed or just have my coffee”* (Student).

Participants described the benefits of having such a third place, such as detaching from their day-to-day stressors and realities, and having a place to take a break. They spoke about the perceived benefits of spending time in the garden, with over half discussing how being in the garden space allowed them to slow down and decompress.

*“it gives you kind of a nice transition period” and serves as “a moment of calm”* (Student)

*“[I]t’s just nice to sort of take the time out for it, to be around these—, to sort of get away from technology and uh sort of the stress of what you’re kind of getting on with, and take some time to watch things grow, and spend some time caring for things, and spending time outside around, that sort of stuff”* (Student).

*“I detach from reality and...it’s almost like brain hygiene...it’s nice”* (Student)

### 3.2. Increased connectedness to nature

Most study participants described how their involvement in the community garden fostered an increased sense of connection to nature, facilitated through their direct engagement with the plants, cultivating their plots, and interactions with local wildlife. They described connectedness as engagements with the community garden that fostered moments of tranquility, alongside an increased sense of purpose. Below two sub-themes under ‘nature connectedness’ are presented: First, sensory engagement as a facilitator of nature connectedness; and second, an increased sense of responsibility.

#### 3.2.1. Sensory engagement as a facilitator of nature connectedness.

Participants discussed the act of gardening as an immersive and multi-sensory experience, detailing textures, scents, sounds, and visuals. They described how these sensory experiences in the garden facilitated feelings of being present in the moment within the garden and connected to life, which in turn fostered an improved sense of well-being. Participants also spoke about how their experiences of seeing wildlife while in the garden created a sense of happiness that in some cases they carried with them, even later on after leaving the garden.

*“[y]ou’re smelling flowers, you’re touching the leaves, you’re outside...that for me is really important...an important part of being alive”* (Student).

*“I think it was that opportunity for me to...let myself connect with nature...each and every moment I was there caring for my plants, I felt good”* (Student).

*“...because you’re working quietly in there, they haven’t noticed me, and I’ve kind of like, you know, there’s a huge hawk or something to be around, and it’s such a wonderful experience, and mentally, that’s just wonderful for you because you walk around going, that made my day…”* (Student).

#### 3.2.2. Sense of responsibility.

Participants described feeling responsibility for their plants as augmenting their sense of connection to nature and cultivating a sense of responsibility to the larger environment. The process of being part of the garden and tending to their plots provided a perspective shift for some participants, who came to view themselves more as caretakers and stewards rather than simply gardeners. They in turn then took a more active role in the garden, and towards broader environmental sustainability initiatives. They also described how their satisfaction from cultivating plants became rooted in the responsibility that they took on, which in turn also created a sense of achievement in sustaining their plants and spurred them to take a more active role in other garden and environmental initiatives. Participants also described how their sense of responsibility developed as they experienced personal growth and confidence in themselves as caretakers through their experience gardening.

*“I think a lot of it is that sense of satisfaction and connection…like that satisfaction of like, you did that, like you watered that, you can see that grow. Like you are quite literally bringing life into the world. I think that’s really satisfying. I think there’s also a satisfaction in like maintaining a responsibility and owing something, like owing things to each other and like owing something to the plants and everything. And I think honoring those responsibilities is really satisfying as well”* (Student).

*“it’s also just nice to participate in, I don’t know how how to explain it because it’s so like it’s almost like it’s meta, like it’s growing these beings, these relatives, these flowers to put into the ground to help the pollinators and all of the other critters that come to these relatives and then help the people around us see the importance in these flowers as well. They’re not just flowers. They’re medicine. They’re their homes to insects”* (Student).

*“I suppose the nice part of it was actually just realizing that at least on this small scale of things…I can actually take care of things and I don’t, with a bit of luck, I don’t kill every plant I touch or something”* (Student).

### 3.3. Empowerment through growing food

For many participants, the act of growing food in the community garden served to both supplement their diet and foster a sense of empowerment through skills and knowledge to be self-sufficient and in control over their food sources. Through gardening, participants gained confidence in their abilities to contribute meaningfully to their own sustenance, making their participation a transformative, empowering experience. This theme includes two sub-themes: 1) empowerment through knowledge on how to grow food and 2) knowledge of, and access to alternative food sources.

#### 3.3.1. Empowerment through knowledge on how to grow food.

Participants described how they developed skills and knowledge that helped them to foster an improved sense of self-reliance. The hands-on experience in growing their own food contributed to feelings of capability and control over their food sources. Participants also described how learning about crop choices, seasonal growing, and sustainable practices contributed to their confidence in their ability to produce food.

Most participants emphasized how developing skills and learning cultivation processes made them feel more empowered and self-sufficient, even though this took time (e.g., for some, many seasons). Participants also spoke about a desire to receive and share knowledge with others, indicating that learning to grow food is an empowering experience that participants value and wish to promote.

Participants also demonstrated agency in their food production, speaking about how they adapted their crop choices to fit personal needs. For example, some chose to grow lettuce to have a source of fresh greens, while others grew herbs because they knew they were hardier. These adaptations reflected a practical understanding of how to make gardening work best for their needs and lifestyle. This knowledge reinforced their confidence in independently managing food production, making sustainable food consumption choices, and being mindful of food waste and how to best mitigate it through their gardening practice.

*“The first year I planted some…tomatoes and then the second year I planted some…lettuce and kale because it was convenient to have…a fresh source of...leafy things. I found in my fridge, they would go bad before I would use them, but in the garden, you can just leave them to… grow and then you just cut off a leaf for…a burger or…a salad when you need it”* (Staff/Faculty).

*“I think it is something where it is like a slower process. Cause [sic] like, I don’t think you can guarantee a certain amount of produce or anything, but like you’re developing that knowledge base and like I think it’s better than not doing it, obviously”* (Student).

*“I also think that it may be a benefit to almost have some sort of like document or something that is a walk through for folks to grow their own like everyday salad or something like that”* (Student).

#### 3.3.2. Alternative food sources: knowledge and access.

Participants described how the garden provided them with an alternative food source, reducing their reliance on commercial food supplies during the growing season and helping them to create independence from external systems for basic needs. By supplementing their groceries with garden-grown produce, participants discussed how they gained a practical sense of independence in their ability to meet their own food needs directly.

*“...there is the opportunity because let me tell you something, I was bringing home a lot of food, so I know firsthand that it contributed to my own food security”* (Student).

*“I don’t know how much it will reduce the cost of groceries, honestly, but definitely has an impact...because you will buy less of the vegetables part of the grocery”* (Student).

*“So despite the size, the tiny size of the plot of land, you can still grow, you know, some vegetables over there, some herbs…it was quite successful. So I was able to provide myself, for example, with tomatoes for a few months because they, you know, kept coming”* (Staff/Faculty).

*“I suppose in a small scale just in terms of, it would allow an additional bit of fresh produce and I mean that— one of the things that I realize I’m an outlier because I did grow more herbs than fruit or vegetables. And, but in terms of as a supplement absolutely…”* (Student).

### 3.4. The garden as a facilitator for community building

Participants described how the garden fostered community building through joint participation and providing a space to reconnect with their campus community, something that was particularly meaningful following COVID-19 pandemic lockdowns and related social isolation. This theme includes two sub themes: 1) fostering connections through shared values and 2) post-pandemic reconnection and social integration.

#### 3.4.1. Fostering connections through shared values.

For most participants, a motivator for joining the garden was to meet like-minded individuals. They described how the social aspects of their garden involvement were impactful for building connections with people with shared values and interests. Participants also spoke about how engaging in the gardening process with others made them feel supported and connected, especially for those new to gardening. The garden space was described as more than just a place, but rather a supportive and non-competitive place for cultivating conversations.

*“Connection being number one. I love connecting with other gardeners and other farmers., I see the value in having access to a place where you can grow something”* (Student).

*“[I]t was nice to meet people...just to support each other, everyone has the same goal in mind—it’s not like “I plan to outgrow you…there’s nothing of that sort. so it’s me, it’s just community garden, community building and I guess just helping each other”* (Staff/Faculty).

*“it was also an opportunity to just connect with people who share similar values. I’ve had a lot of really good conversations with people that I meet in the garden, umm who are like, hey, you know, what brings you here? Umm we talk about different things, I can share tips with them about, you know, gardening at home or, you know, nature on campus and that sort of thing”* (Student).

#### 3.4.2. Post-pandemic reconnection.

Some participants also spoke about how the garden helped them to get involved in communal sustainability initiatives following the COVID-19 pandemic lockdowns. They described how the community garden helped to facilitate social interactions and community-building by providing a space to work collectively on campus with others with shared interests. Participants also described how participating in the garden over multiple years fostered connections over time and supported integration within the campus community. Ultimately, the garden provided a form of connection and reconnection, building from participants’ shared interests and passion for sustainability.

*“this year was the first time I went out on a space and so. I guess, partly it was just coming out of the pandemic and not being able to actually be on campus for the longest time. Just to, getting that bit more encouragement to actually come to campus, and work there and also just to have a bit of a chance to, well, hopefully, to, just you know, participate in the work that the community garden is doing just in terms of building awareness of the usefulness of these spaces and just the community aspect of it in just being in the community working together on that sort of sustainability initiative”* (Student).

*“...I want to get more involved…sustainability initiatives and…environmental stuff and…also make friends and…maybe connect [with] new people and so I think that was part of it that first year—cuz [sic] I had really just moved to London because I had lived in [redacted location] to do work for the beginning of my master’s because it was during COVID so I don’t have any classes in person. So I’d only been in London like 3 or 4 months at that point. And I was looking to get more involved and try something new. And then I think this time with the group garden, I was…more secure in my friendships and…it was more about…doing something together”* (Student).

## 4. Discussion

The community garden at Western University in London, Ontario, Canada has stood as a beacon of sustainability and eco-conscious innovation amidst escalating mental health, environmental, and food security concerns [27]. This study explored and documented the experiences of participants in the campus community garden, identifying and exploring their perceived health and well-being benefits of garden participation. The thematic analysis culminated in a rich, detailed, and complex account of the data, underscoring the significance and contextual relevance of the study’s findings in relation to third places, connectedness to nature, and community engagement. This study found that the community garden served as a form of third place for participants, positively impacted their perceived connection to nature, empowered participants through the act of growing food, and acted as a facilitator for community building. However, some participants did experience different benefits from participating in the garden, as some participants were volunteers tending to the same plot because of their membership in a graduate student organization, compared to individuals who maintained their plot solely on their own and thus reaped the benefit of being the sole person to reap the benefits of a full harvest.

We build upon a growing literature on ‘third places’ and nature-based interventions, with this study identifying that the community garden served as a ‘third place’ for participants, which facilitated social interactions, community building and social capital among participants. Third places are public settings outside the home and work or school that are accessible and offer a space for individuals to connect and socialize [31]. Research suggests third places can facilitate social inclusion, reduce isolation, and contribute to individual well-being by offering informal, low-pressure environments for interaction [32]. These places can be especially important for individuals who may lack strong social networks elsewhere, functioning as sites of belonging and shared purpose [33]. This aligns with Duff’s [34] concept of *enabling places*, which describes places that support well-being by providing material, social, and affective resources. The garden, through its accessibility, shared purpose, and emotional impact, reflects many of these characteristics. Building upon Duff’s concept of enabling places, Hope et al. (2023) propose the notion of “enabling landscapes,” emphasizing that a network of interconnected places collectively supports individuals’ well-being and recovery by providing diverse material, social, and affective resources [35]. As the amount and availability of third places decline in modern society, it is increasingly important for spaces such as community gardens to serve as neutral spaces that are accessible and accommodating to all who visit, offering a place to engage with others and build connections [36]. Community garden participants can build social capital through shared activities such as growing and eating food, which are sociable and open to people of all ethnicities, ages, and socio-economic backgrounds [10]. Further, community gardens build social capital by strengthening links between authorities and institutions that enable resources to be accessed by those within the garden [10]. As found in this study, some participants mentioned the impact of the COVID-19 pandemic lockdowns on the lack of connection they had to their university community. The COVID-19 pandemic contributed to higher levels of mental distress, making the garden an important place for participants to cultivate connections with others and integrate into the campus community [36]. This is especially important within the context of the mental well-being challenges faced by this population, which were worsened by the COVID-19 pandemic across outcomes of wellbeing, school connectedness, and social relationships [37]. The mental health and well-being benefits of gardening may stem from the act of interacting and having tasks to do in nature, which provide distraction and give a perspective on life [38]. This is supported by what participants shared in interviews on how gardening provided them with a new perspective and way to connect with the natural world. These findings are also supported by the biophilia hypothesis, which suggests that humans have an innate and biological affinity for the natural world, and it is a biologically based need to focus on life and lifelike processes [39,40].

This study also found that the campus community garden participation fostered a deeper connection with nature amongst participants. Participants described this connection as offering moments of calm, opportunities to slow down, and multi-sensory experiences that cultivated mindfulness and presence. This finding dovetails with an extensive literature that suggests connectedness to nature can support wellbeing, including a calming effect on their emotions [41], improving eudaimonic wellbeing (happiness) [42], reducing anxiety [43], improving household pro-environmental behaviours [44], and improving social connectedness [45].

Many participants described how the community garden allowed them to decompress, detach from daily stressors, and experience moments of calm and clarity. These findings align with theories such as Attention Restoration Theory, which proposes that natural environments can relieve mental fatigue by offering immersive and restorative experiences [46]. Time in nature may support individuals to be away from everyday stress, experience expansive spaces, participate in activities that are more compatible with our intrinsic motivations, and experience fascinating stimuli [44,45]. The sensory connections to nature reported by participants such as the smells, sounds, and feel of working in the dirt were described as engaging, meditative, and creating a sense of wonderment for participants [46].

Furthermore, this study highlighted participant feelings of empowerment as a result of gaining the knowledge to grow their own food, and their ability to provide themselves with an alternative food source. In an era of concurrent cost of living and mental health crises, many university students are experiencing greater financial precarity, including increased food insecurity, leading to a rise in food bank usage on campuses [47]. Community gardens on university campuses, could be an effective tool for addressing these challenges. A qualitative study at New Mexico State University explored the role of their campus community garden in addressing food insecurity and found participation in the garden not only provided access to fresh produce but also empowered students by teaching them how to grow their own food, thereby enhancing their food security skills [48]. Similarly, the Rachel Carson Council reports that campus gardens not only help address student food insecurity but also equip students with practical skills in sustainable agriculture and deepen their understanding of food systems, empowering them to advocate for food justice [49]. The United Nations Sustainable Development Goals encompass many areas of health and well-being and community gardens may help post-secondary institutions by aligning with Sustainable Development Goal 2, “[e]nd hunger, achieve food security and improved nutrition and promote sustainable agriculture” [50]. Findings add to this literature in the Canadian context, suggesting that community gardens can address immediate food needs by expanding the sources of food available to participants, as well as educating participants on sustainable agricultural practices, providing short-term solutions and long-term educational benefits. However, the impact of seasonality on growing seasons is an important consideration for the extent to which food security and nutrition needs may be met, as the outdoor garden growing season is limited by the winter months in Canada. In this study, seasonality also influenced participation patterns, particularly among undergraduate students who were less likely to be involved during the summer months when many returned home for academic breaks. As a result, participation tended to be higher among staff, faculty, and graduate students who remained in the area year-round. Future initiatives could address this challenge by exploring the use of greenhouses or indoor growing spaces to extend the growing season and enable greater continuity of student engagement throughout the year.

### 4.1 Future directions

Continued support for campus community garden plots depend on the support of university stakeholders. Studies have shown that effectively communicating the value of these gardens to university stakeholders is crucial for their sustainability and impact [46,51]. This highlights the importance of stakeholder support through various channels such as alumni contributions and community engagement, continuing to raise awareness about the importance of these spaces on student health and well-being [46,51].

Given the findings of this study, future efforts could focus on institutionalizing these gardens as part of campus wellness strategies and expanding access for a wider range of students. For example, implementing garden orientation programs or integrating garden activities into academic curricula may enhance visibility and participation. Additional strategies such as combining community garden participation with campus mental health programming could also provide a means of solidifying the role of campus community gardens within post-secondary settings. These strategies align with student wellness goals as well as embed the garden within the broader network of campus life.

### 4.2 Limitations

This study was conducted on one university campus with a small group of garden participants, which may limit the generalizability of the results to other institutional or geographical contexts. Participants in the study may have been more likely to participate in this research due to stronger feelings about nature, the environment, and the garden potentially introducing a selection bias. The study relied on self-reported data through interviews, which may be influenced by memory recall or social desirability. Future research would benefit from diverse geographical sites, larger and broader participant samples, and mixed methods approaches to strengthen the validity of the findings.

## 5. Conclusion

The purpose of this study was to examine the experiences and perceived benefits of participating in a campus community garden as a nature-based health intervention. Findings document the multifaceted ways in which campus community gardens may serve as a third place. Further, benefits shared by study participants included improved mental well-being, social connection, increased sense of environmental responsibility, and food empowerment. They also reported an increased sense of connectedness to nature through sensory engagement and caretaking, as well as increased self-efficacy and autonomy gained from cultivating food. An additional benefit was the space for community building and social interaction, which was described as especially meaningful in the aftermath of the COVID-19 pandemic lockdowns. These findings suggest the potential for campus community gardens to serve as accessible, cost-effective interventions that promote mental health, food security, and social well-being in post-secondary environments.
